# Complete Genome Sequence of an Aldoxime Degrader, Bacillus sp. OxB-1

**DOI:** 10.1128/genomeA.00025-15

**Published:** 2015-02-26

**Authors:** Takuya Yamaguchi, Yasuhisa Asano

**Affiliations:** a Biotechnology Research Center and Department of Biotechnology, Toyama Prefectural University, Imizu, Toyama, Japan; b Asano Active Enzyme Molecule Project, ERATO, JST, Imizu, Toyama, Japan

## Abstract

Bacillus sp. OxB-1 has been characterized as a strain that produces a new enzyme, aldoxime dehydratase, which catalyzes the dehydration of aldoxime to form nitrile. Here, its complete genome sequence (3,594,618 bp, with a GC content of 47.85%), comprising a circular chromosome, is announced.

## GENOME ANNOUNCEMENT

Bacillus sp. OxB-1 was isolated as a (*Z*)-phenylacetaldoxime-degrading bacterium from local soil in Japan (1). The strain metabolizes (*Z*)-phenylacetaldoxime into phenylacetic acid via phenylacetonitrile by a combination of aldoxime dehydratase (Oxd) and nitrilase (2). Nitrile, an intermediate of the pathway, is used extensively in the chemical industry (3). The chemical synthesis of nitrile is performed under harsh conditions and requires toxic chemicals (4). Because the enzymatic reaction is environmentally friendly, recombinant Oxd has been applied to nitrile production from aldoxime (5, 6). In addition, the combination of Oxd and a plant aldoxime-forming cytochrome P450, CYP79A2, has enabled the fermentative production of phenylacetonitrile from l-phenylalanine (7). However, the biological aspect of the aldoxime-degrading pathway of the strain has not been elucidated. The complete genome of OxB-1 was sequenced with the aim of gaining a better understanding of the pathway.

The complete genome of OxB-1 was sequenced by a shotgun strategy using HiSeq 2000 (Illumina, Hayward, CA, USA), which produced paired-end and mate-paired reads totaling ~800 Mb with approximately 220-fold coverage of the genome. Genome sequence data were processed and assembled into nine scaffolds using SOAPdenovo version 1.05 (8). Gaps between scaffolds were manually closed by PCR and Sanger sequencing. Finally, this assembly process produced a large scaffold circular chromosome. The genome sequence was annotated using the Microbial Genome Annotation Pipeline, which is a combination of MetaGeneAnnotator (9) for protein coding sequence (CDS) prediction, RNAmmer (10) for rRNA prediction, tRNAscan-SE (11) for tRNA prediction, and BLAST (12) for a homology search.

The genome of OxB-1 is 3,594,618 bp long and has a GC content of 47.85%; it contains 3,587 CDS, 7 rRNA operons, and 83 tRNA genes. In total, 3,234 CDS matched known genes (90% of all CDS). A gene encoding AraC-like transcriptional regulator was found in the gene cluster containing Oxd and nitrilase genes. It seems to regulate the expression of Oxd and nitrilase, given that the expression is induced by (*Z*)-phenylacetaldoxime (2).

Aldoxime is biosynthesized from amino acids by cytochrome P450s or flavin monooxygenases in plants, insects, and bacteria (13–15). CDS encoding a cytochrome P450 and four flavin monooxygenases were found in the genome as candidate genes. The complete genome of OxB-1 will aid in the identification of aldoxime-forming enzyme(s) in Bacillus sp. OxB-1.

### Nucleotide sequence accession numbers.

This whole-genome shotgun project has been deposited in DDBJ/EMBL/GenBank under the accession no. AP013294. The version described in this paper is the first version, AP013294.1.
